# Association of H-Type Hypertension with Stroke Severity and Prognosis

**DOI:** 10.1155/2018/8725908

**Published:** 2018-09-09

**Authors:** Tan Li, Jiajia Zhu, Qi Fang, Xiaoyu Duan, Mingzhi Zhang, Shanshan Diao, Yun Zhou, Si Yang, Yan Kong, Xiuying Cai

**Affiliations:** ^1^Department of Neurology, The First Affiliated Hospital of Soochow University, No. 899, Pinghai Road, Suzhou, Jiangsu 215006, China; ^2^Department of Epidemiology & Biostatistics, Jiangsu Key Laboratory of Preventive and Translational Medicine for Geriatric Diseases, School of Public Health, Medical College of Soochow University, Suzhou, Jiangsu 215006, China

## Abstract

*Background. *The correlation between H-type hypertension and acute ischemic stroke remains uncertain.* Objective.* The present study was designed to explore the possible relationship between H-type hypertension and severity and prognosis of acute ischemic stroke.* Method.* We included 372 patients with acute ischemic stroke and divided them into four groups: H-type hypertension group, simple hypertension group, simple hyperhomocysteinemia (HHcy) group, and the control group. NIHSS score was measured at both admission and two weeks later. mRS score, stroke recurrence, cardiovascular event, or all-cause mortality was recorded at 3-month and 1-year follow-up.* Result.* The results showed that the NIHSS score on admission in the H-type hypertension group (6.32 ± 5.91) was significantly higher than that in the control group (3.97 ± 3.59) (P < 0.05), while there was no obvious association between H-type hypertension and NIHSS score after 2-week treatment (P = 0.106). Endpoint events incidence in H-type hypertension group was the highest; however, in the cox regression model of multiple factor analysis, H-type hypertension was not an independent risk factor.* Conclusion.* H-type hypertension may result in early functional deterioration and higher incidence rate of endpoint events but not act as an independent risk factor.

## 1. Introduction

Ischemic stroke has become the leading cause of disability and mortality in China [1]. In addition, it remains a major cause of functional impairment and increases the burden on those who suffer a stroke, their families, other caregivers, and the national economy.

Homocysteine (Hcy) is a sulfur-containing amino acid mainly derived from methionine. Recently, mounting evidences have shown that hyperhomocysteinemia (HHcy) might be an independent risk factor of early neurological deterioration [2] and poor outcome [3, 4] in patients with acute ischemic stroke, which is possibly through proinflammatory response [5], neurotoxicity, endothelial dysfunction, and atherosclerosis mechanisms [6].

H-type hypertension, which refers to concurrence of primary hypertension and HHcy, is a special hypertension type. Approximately 75% of the hypertensive patients simultaneously have HHcy in China [7]. Previous studies suggested that H-type hypertension could be positively related to the risk of cardio-cerebrovascular [8, 9]. However, whether it could be regarded as an independent predictor of severity and prognosis of ischemic stroke has not been fully clarified. Thus, this study was designed to explore the possible relationship between H-type hypertension and acute ischemic stroke.

## 2. Methods and Materials

### 2.1. Study Design and Subjects

The present study was conducted in Stroke Center of First Hospital Affiliated to Soochow University and included 372 patients who had been diagnosed with acute ischemic stroke according to the World Health Organization (WHO) criteria [10] and confirmed by brain computed tomography (CT) or brain magnetic resonance imaging (MRI) from October 2015 to February 2017. The patients who were >18 years of age had no cognitive impairment and no serious consciousness were included. Exclusion criteria for the study were transient ischemic attack (TIA), epilepsy, severe renal dysfunction, previous history of hypothyroidism, trauma, recent surgery, other significant acute medical illness, and incomplete information. At least 2 trained neurologists from our stroke center evaluated the clinical features and diagnostic test results. The participants were divided into four groups: H-type hypertension group, simple hypertension group (patients with elevated blood pressure only), simple HHcy group (patients with high plasma level of Hcy only), and the control group who had neither hypertension nor HHcy. All patients gave informed consent to join in and all data were analyzed anonymously. Ethical approval for this study was obtained from the ethics committees of the First Hospital Affiliated to Soochow University.

### 2.2. Clinical Information

Sociodemographic information on age (calculated according to the ID birth date), gender, education level, marital status were collected. Education level included “under junior”, “senior/vocational high school”, “college degree or above”. Marital status was divided into “unmarried”, “married”, “divorced”, and “spouse”. Lifestyle factors include smoking and alcohol consumption; past medical history, family history, disease history of hypertension history, diabetes history, stroke history, hyperlipidemia history, and coronary heart disease history were obtained. “Smoking consumption” was defined as having 10 or more cigarettes per day for more than one year [11] or smoking cessation <5 years [12]. “Alcohol consumption” was regarded as drinking more than 2 units per day. Hypertension was recorded as having previous history of hypertension or systolic blood pressure equal to or higher than 140 mmHg and/or diastolic blood pressure equal to or higher than 90 mmHg for two times in the quiet condition. Diabetes mellitus was confirmed as having previous history of diabetes, or fasting serum glucose > 7.0 mmol/L after admission or postprandial 2 h plasma glucose > 11.1 mmol/L. Patients who have elevated level of one of total cholesterol, low-density lipoprotein, and triglyceride were known as hyperlipidemia. Full neurological examination, brain CT, or MRI scan and carotid ultrasonography were also recorded.

### 2.3. Blood Collection and Indexes Determination

Venous blood samples from patients were collected on an empty stomach the second day of hospitalization for further determination including hemoglobin contents, total cholesterol (TC), low-density lipoprotein cholesterol (LDL-c), high-density lipoprotein cholesterol (HDL-c), triglyceride (TG), uric acid, glucose, and so on. The serum Hcy levels were measured within 24 h of hospitalization using enzymatic cycling method. HHcy was defined as Hcy concentration >=12.0 umol/L.

### 2.4. Outcomes and Follow-Up

National Institutes of Health Stroke Scale (NIHSS) was measured both at admission and after two weeks to evaluate the severity of stroke outcome. NIHSS ranged from 0 to 42, and higher score on NIHSS represented more severe stroke. The modified Rankin Scale (mRS) was measured to assess the patients' functional prognosis, with a span from 0 to 6. We set 0-2 as favorable prognosis and 3-6 as poor outcome. We followed up the enrolled patients 3 month and 1 year after discharge. The primary endpoint event of the present study was stroke recurrence and the secondary endpoint events were cardiovascular event including coronary heart disease, myocardial infarction, heart failure, coronary artery reconstruction, cardiopulmonary resuscitation, or all-cause mortality (death for any cause). Stroke recurrence referred to the following: (1) original neurologic impairment improved or disappeared, then new ipsilateral or contralateral symptoms emerged for at least 24 hours; (2) original neurologic impairment aggravated with exclusion of progressive stroke; (3) the above situation was confirmed by head CT or MRI. The follow-up was conducted by 2 trained neurological doctors who were blinded to the baseline information and grouping situation.

### 2.5. Statistical Analysis

Statistical analysis was performed with SPSS13.0 (SPSS, Inc., Chicago, IL, USA). Normally distributed continuous variables were reported as mean ± Standard Deviation (SD), while deviation and categorical variables were presented as frequency and percentage. Kolmogorov–Smirnov was used for the test of normal distribution of quantitative data and Levene's test was used to test homogeneity of variance. T test or one-way ANOVA was performed to compare the distribution of quantitative data. *χ*^2^ test was used to compare the distribution of classification index. The HRs and 95% CIs for the risk factors associated with the stroke endpoint events were investigated with Cox proportional hazards regression with adjustment for age, sex, hypertension, diabetes, cardiac disease, blood pressure, triglycerides, glucose, uric acid, high sensitivity C-reactive protein (Hs-CRP), Hcy, and H-type hypertension. Cumulative risk of stroke recurrence, cardiovascular events, and all-cause death were performed using Kaplan–Meier survival curve. 2-tailed values of p < 0.05 were considered to be statistically significant in the present study.

## 3. Results

### 3.1. General Clinical Characteristics of the Study Population

A total of 372 (240 males and 132 females) patients with acute ischemic stroke were finally included and the baseline information of the cases were listed in Table 1. In the present study, the first top risk factor of ischemic stroke was hypertension (68.3%) followed by age, gender, HHcy, Hs-CRP, cardiac disease, hyperlipidemia, diabetes, and hypeluricemia in turn. The most common stroke subtype observed in our study was large artery atherosclerosis (LAA) (53%), in turn followed by small artery occlusion (SAO), cardioembolism (CE), stroke of other determined etiology (SOE), and stroke of undetermined etiology (SUE).

Table 2 summarized the clinical features of patients with H-type hypertension. Of the 254 hypertensive patients enrolled in our study, half had H-type hypertension and the other half had non-HHcy. When compared the control group to the H-type hypertension group, age, systolic pressure (131.83 ± 9.63 versus 147.98 ± 17.29), uric acid (282.88 ± 79.00 versus 345.72 ± 113.72), and Hcy (345.72 ± 113.72 versus 21.84 ± 25.20) were significantly different between the two groups (all P < 0.01). When it comes to the simple hypertension group and H-type hypertension group, the elderly and higher Hs-CRP (4.21 ± 4.62 versus 5.91 ± 5.21) levels were observed in H-type hypertension group (P < 0.05), and also gender, cardiac disease (13.4% versus 26.8%), and uric acid (285.40 ± 77.28 versus 345.72 ± 113.72) had significant difference between the two groups (P<0.01). Diabetes (5.6% versus 21.3%) had a significant association with H-type hypertension compared with simple HHcy group; besides, H-type hypertension further elevated the level of uric acid compared with the simple HHcy group (295.5 ± 88.55 versus 345.72 ± 113.72) (all P < 0.01).

### 3.2. The Association of Severity of Stroke with H-Type Hypertension

The severity of stroke was assessed by using the NIHSS score, which was obtained both on admission and 2 weeks after treatment. Our results showed that the score on admission in the H-type hypertension group (6.32 ± 5.91) was significantly higher compared with that in the normal group (3.97 ± 3.59) (P < 0.05), while we did not observe the difference of baseline NIHSS between the H-type hypertension group and simple hypertension group or simple HHcy group. In addition, there is no obvious association between H-type hypertension and NIHSS score after 2 weeks treatment (P = 0.106). These findings were presented in Table 3. The study suggested that acute ischemic stroke patients with H-type hypertension were at an increased risk for early neurological deterioration.

### 3.3. The Association of Prognosis of Stroke with H-Type Hypertension

mRS score was used to present the long-term neurological functional recovery of the stroke patients. Our results showed that mRS scores were 2.39 ± 1.52 and 1.77 ± 1.21 at 90-day follow- up, 1.98 ± 1.43 and 1.34 ± 1.20 at one-year follow-up in H-type hypertension group and control group, respectively. The mRS score was significantly different between the H-type hypertension group and the control group (P < 0.05). These results were shown in Table 4.

**Figure 1:**
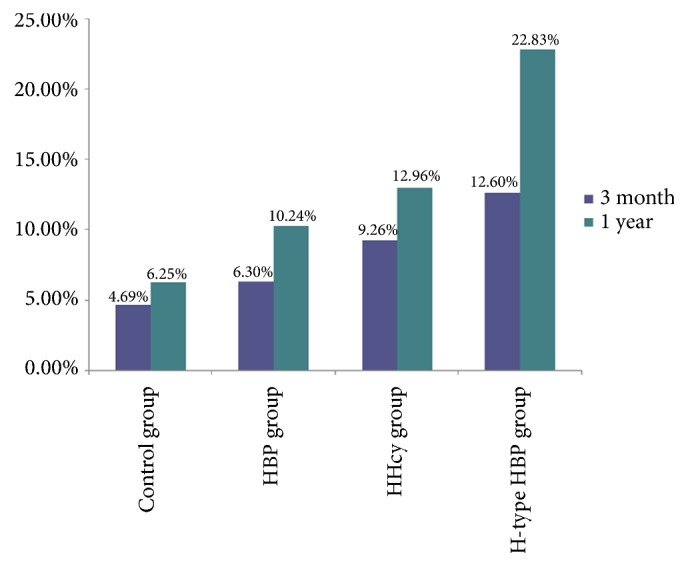
Incurrence of endpoint events at 90-day and 1-year follow-up among groups.

In the present study, stroke endpoint events were defined as stroke recurrence, all cardiovascular diseases and all-cause death. During 1 year of follow-up, a total of 33 (8.87%) recurrent stroke, 8 (2.15%) cardiovascular events, and 12 (3.22%) all-cause deaths were recorded in all the enrolled patients. 16 patients (12.6%) with H-type hypertension reached the combined endpoint events compared with 3 (4.69%) patients in the control group, 8 (6.30%) patients in the simple hypertension, and 5 (9.26%) patients with simple HHcy during the 90-day follow-up period. During the 1-year follow-up period, patients reached to the combined endpoint events were 29 (22.83%), 4 (6.25%), 13 (10.24%), and 7 (12.96%) in the H-type hypertension group, control group, simple hypertension group, and simple HHcy group, respectively. (Figure 1)

**Figure 2:**
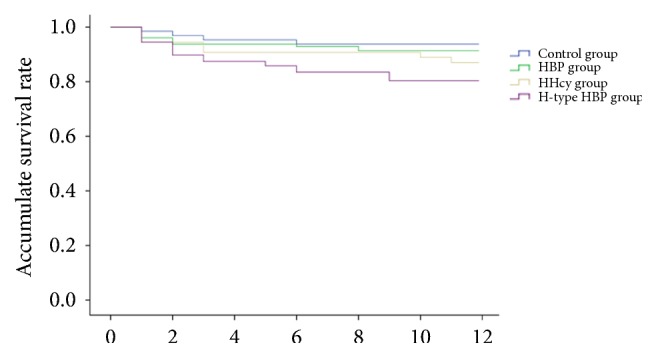
Cumulative survival curve of the stroke patients among groups.

Kaplan–Meier survival analysis showed that when we compared all acute ischemic stroke patients at 1-year period, patients with H-type hypertension had the lowest cumulative survival, as shown in Figure 2. A number of predictors of stroke endpoint events were shown in Cox regression model (Tables 5 and 6). Single factor analysis indicated that age, systolic pressure, cardiac diseases, glucose, Hs-CRP, and H-type hypertension were contributed to the high incidence of endpoint events after stroke. However, after the multivariate adjustment, endpoint events incidence of stroke was significantly related to age (HR: 2.740, 95% CI: 1.236-6.073), the glucose (HR: 2.047, 95% CI: 1.146-3.655), and cardiac diseases (HR: 2.041, 95% CI: 1.082-3.849). The results indicated that H-type hypertension was not an independent risk factor of endpoint events incidence after stroke.

## 4. Discussion

Stroke is one of the major causes of increased morbidity and mortality all over the world. In China, the prevalence of hypertension is high and HHcy is one of the common risk factors for hypertension in Chinese populations. Recently, H-type hypertension has become a research hotspot in the area of cerebral-cardiovascular diseases.

In the present study, higher NIHSS score and mRS score were noted in H-type hypertension group, compared with that in the non-H-type hypertension group, which implied poor short-term and long-term outcomes in H-type hypertension patients. Moreover, incidence of endpoint events, especially stroke recurrence was higher in H-type hypertension patients compared with other groups, together with the lowest cumulative survival in this group. Several researches had come to the similar results. Chongke Zhong et al. found that enrolled patients with H-type hypertension were at the highest risk of poor outcome among all participants [13]. Another study also found that H-type hypertension (OR = 2.988, 95% CI: 1.162–7.686) was an independent risk factor for recurrence stroke [14]. However, in Cox regression analysis, after the multivariate adjustment of age, glucose, and cardiac diseases, our results did not suggest that H-type hypertension could be an independent risk factor of endpoint events after stroke.

Some recent studies suggested that hypertension and HHcy might have a certain synergistic effect [15–17]. Graham et al. found that, in patients with H-type hypertension, the incidence of cardiovascular diseases was about five times higher than that of patients with simple high blood pressure; thus they speculated that the synergistic effect between hypertension and HHcy [18]. Hui Pang studied 2258 hospitalized patients with hypertension to evaluate the association of H-type hypertension and stroke incidence. They noticed a remarkable increase of stroke morbidity in patients with H-type hypertension compared with that in non-H-type hypertension group, and both SBP and DBP had a positive correlation with stroke morbidity for H-type hypertensive patients [19]. The CSPPT discovered that a combined antihypertension (enalapril) and homocysteine-lowering therapy could significantly reduce the risk of first stroke by 21% (HR, 0.79; 95% CI: 0.68-0.93) compared to those patients with enalapril alone [20].

The primary mechanisms of H-type hypertension acting on stroke remained unclear. Guo G found that the risk of plaques occurrence in patients with H-type hypertension was 1.63 times of patients with simple (or isolated) systolic hypertension. They further discovered that high homocysteine concentration might aggravate the oxidative stress in hypertension to produce contributory effects on vascular impairment [21]. Xu H et al. found that reduction of CD4+T-cell percentage might be an important cause of immune disorders of H-type hypertension patients. According to their results, Hcy oxidized to peroxide, leading to immune status disorders and T-cell subsets imbalance. The decrease of blood pressure protective Treg cells will aggravate hypertension and vascular injury [22]. Furthermore, there is growing evidence of the association between H-type hypertension and type 2 diabetes (T2DM) [20], which may promote insulin resistance and increase the risk of death in patients with T2DM [23, 24]. Thus, H-type hypertension may result in early functional deterioration and higher incidence rate of endpoint events via aggravating other risk factors rather than acting as an independent risk factor.

This study is to investigate the correlation between H-type Hypertension and acute ischemic stroke. However, this study has several limitations. The Hcy concentration was measured only at one time-point within 24 hours after stroke onset, and we have no data on possible changes in Hcy plasma level or blood pressure during long-term clinical follow-up period. Therefore, these relationships were not examined on the 90-day and 1-year clinical outcome. Subsequently, several studies indicated that medicines such as aspirin, clopidogrel, statins, and hypotensive drugs may decrease inflammatory mediator levels and affect the results, and some medicines may affect Hcy metabolism. However, we did not record patients' medications at baseline. Additionally, the relatively small sample size was used in this study, and the follow-up period was relatively short, which remained as a limitation on overall assessment of the results.

In conclusion, our study indicated that H-type hypertension may cause increased susceptibility to poor outcome and prognosis among acute ischemic stroke patients, which deserved further prevention measures.

## Figures and Tables

**Table 1:** The most common risk factors of stroke in our study.

Variables	
Age (year), n (%)	
	129 (34.7%)
≥60	243 (65.3%)
Gender, n (%)	
Male	240 (64.5%)
Female	132 (35.5%)
Hypertension, n (%)	254 (68.3%)
Diabetes melitus, n (%)	74 (19.9%)
Cardiac diseases, n (%)	80 (21.5%)
Hyperlipidemia, n (%)	75 (20.2%)
HHcy, n (%)	181 (48.7%)
High level of urid acid, n (%)	48 (12.9%)
High level of Hs-CRP, n (%)	158 (42.5%)

**Table 2:** The clinical features of patients with H-type hypertension or without H-type hypertension.

Variables	Control group (n = 64)	HBP group (n = 127)	HHcy group (n = 54)	H-type HBP group (n = 127)	*χ*2/F	P
Age	57.23 ± 13.87	63.97 ± 12.05^**∗**^	64.85 ± 12.20	67.37 ± 13.02^**∗∗**#^	12.115	0.000
Male	40 (62.5%)	69 (54.3%)	35 (64.8%)	96 (75.6%)^**##**^	12.675	0.005
Diabetes melitus	7 (10.9%)	37 (29.1%)^**∗∗**^	3 (5.6%)^**##**^	27 (21.3%)^ΔΔ^	17.141	0.001
Cardiac diseases	10 (15.6%)	17 (13.4%)	13 (24.1%)	34 (26.8%)^**##**^	8.470	0.037
Systolic pressure	131.83 ± 9.63	149.28 ± 18.82^**∗∗**^	138.85 ± 16.54^**##**^	147.98 ± 17.29^**∗∗**ΔΔ^	19.634	0.000
Diastolic pressure	79.02 ± 9.54	85.69 ± 14.22^**∗∗**^	78.61 ± 10.67^**##**^	83.15 ± 13.13	6.087	0.000
Tempeture (°C)	36.54 ± 0.33	36.54 ± 0.32	36.61 ± 0.37	36.64 ± 0.50	1.567	0.197
TC (mmol/l)	4.07 ± 0.90	4.28 ± 1.03	3.89 ± 0.80	4.25 ± 1.19	2.265	0.081
TG (mmol/l)	1.21 ± 0.43	1.73 ± 1.30^**∗∗**^	1.16 ± 0.58^**##**^	1.52 ± 1.07	5.927	0.001
HDL-C (mmol/l)	1.23 ± 0.27	1.25 ± 0.38	1.25 ± 0.29	1.22 ± 0.32	0.156	0.926
LDL-C (mmol/l)	2.41 ± 0.70	2.54 ± 0.89	2.27 ± 0.72	2.54 ± 0.86	1.767	0.153
Glucose (mmol/l)	5.74 ± 1.89	6.82 ± 2.81^**∗**^	5.42 ± 1.16^**##**^	6.22 ± 2.30	6.209	0.000
Urid acid (U/L)	282.88 ± 79.00	285.40 ± 77.28	295.5 ± 88.55	345.72 ± 113.72^**∗∗**##ΔΔ^	11.306	0.000
Hs-CRP (mg/L)	4.14 ± 4.82	4.21 ± 4.62	5.06 ± 5.04	5.91 ± 5.21^**#**^	3.139	0.025
Hcy (umol/l)	7.99 ± 2.51	8.35 ± 2.39	23.33 ± 21.83^**∗∗**ΔΔ^	21.84 ± 25.20^**∗∗**##^	21.273	0.000

Note: ^**∗∗**^P < 0.01, ^**∗**^P < 0.05 versus control group, ^**##**^P < 0.01, ^**#**^P < 0.05 versus simple hypertension group, ^ΔΔ^P < 0.001, ^Δ^P < 0.005 versus simple HHcy group. HBP: hypertension.

**Table 3:** Comparison of NIHSS score on admission and 2 weeks after treatment among groups.

Variables	n	NIHSS score On admission	NIHSS score 2 weeks after treatment
Control group	64	3.97 ± 3.59	2.80 ± 3.18
HBP group	127	5.39 ± 5.44	4.37 ± 6.45
HHcy group	54	6.15 ± 6.14	5.22 ± 7.79
H-type HBP group	127	6.32 ± 5.91^*∗*^	5.20 ± 7.72
F value		2.898	2.052
P value		0.031	0.106

Note: ^**∗**^P < 0.05 versus control group. HBP: hypertension.

**Table 4:** Comparison of mRS score at 90-day and 1-year follow-up among groups.

Variables	n	mRS score at 90 days	mRS score at 1 year
Control group	64	1.77 ± 1.21	1.34 ± 1.20
HBP group	127	2.05 ± 1.43	1.59 ± 1.20
HHcy group	54	2.20 ± 1.47	1.76 ± 1.58
H-type HBP group	127	2.39 ± 1.52^*∗*^	1.98 ± 1.43^*∗*^
F value		2.994	3.743
P value		0.031	0.011

Note: ^*∗*^P < 0.05 versus control group. HBP: hypertension.

**Table 5:** Cox regression analysis of risk factors related to endpoint events of ischemic stroke.

	Single factor analysis		Multiple analysis	
Risk factors	HR	95% CI	HR	95% CI
Age	3.137	1.497-6.654	2.740	1.236-6.073
Gender	0.797	0.443-1.432	0.793	0.422-1.492
Diabetes melitus	0.548	0.248-1.212	0.641	0.374-1.296
Cardiac diseases	2.438	1.390-4.275	2.041	1.082-3.849
Systolic pressure	1.807	1.037-3.149	1.494	0.820-2.722
Diastolic pressure	1.297	0.682-2.468	1.322	0.656-2.665
Total cholesterol	1.763	0.429-7.244	1.057	0.233-4.807
Triglycerides	0.975	0.502-1.893	1.121	0.531-2.366
LDL	1.122	0.650-1.938	1.046	0.583-1.877
Glucose	2.075	1.211-3.556	2.047	1.146-3.655
Uric acid	1.063	0.480-2.354	0.793	0.350-1.795
Hs-CRP	2.174	1.254-3.771	1.531	0.855-2.740

Note: stroke endpoint events were defined as stroke recurrence, all cardiovascular diseases, and all-cause death.

**Table 6:** Cox regression analysis of H-type hypertension related to endpoint events of ischemic stroke.

	Single factor analysis		Multiple factor analysis	
Variables	HR	95% CI	HR	95% CI
control	1.000		1.000	
simple hypertension	1.009	0.252-4.035	0.819	0.202-2.316
Simple HHcy	1.876	0.298-7.070	1.669	0.441-6.312
H-type hypertension	3.890	1.198-12.634	2.694	0.817-8.883

Note: stroke endpoint events were defined as stroke recurrence, all cardiovascular diseases, and all-cause death.

## Data Availability

Data supporting the results in the article can be found, including, where applicable, hyperlinks to public datasets analyzed or generated during the study.
